# Stress-Induced Polyploid Giant Cancer Cells: Unique Way of Formation and Non-Negligible Characteristics

**DOI:** 10.3389/fonc.2021.724781

**Published:** 2021-08-30

**Authors:** Yanwei Song, Yucui Zhao, Zheng Deng, Ruyi Zhao, Qian Huang

**Affiliations:** ^1^Shanghai Key Laboratory of Pancreatic Diseases, Shanghai General Hospital, Shanghai Jiao Tong University School of Medicine, Shanghai, China; ^2^Cancer Center, Shanghai General Hospital, Shanghai Jiao Tong University School of Medicine, Shanghai, China

**Keywords:** polyploidization, stress, endoreplication, polyploid giant cancer cells, neosis

## Abstract

Polyploidy is a conserved mechanism in cell development and stress responses. Multiple stresses of treatment, including radiation and chemotherapy drugs, can induce the polyploidization of tumor cells. Through endoreplication or cell fusion, diploid tumor cells convert into giant tumor cells with single large nuclei or multiple small nucleuses. Some of the stress-induced colossal cells, which were previously thought to be senescent and have no ability to proliferate, can escape the fate of death by a special way. They can remain alive at least before producing progeny cells through asymmetric cell division, a depolyploidization way named neosis. Those large and danger cells are recognized as polyploid giant cancer cells (PGCCs). Such cells are under suspicion of being highly related to tumor recurrence and metastasis after treatment and can bring new targets for cancer therapy. However, differences in formation mechanisms between PGCCs and well-accepted polyploid cancer cells are largely unknown. In this review, the methods used in different studies to induce polyploid cells are summarized, and several mechanisms of polyploidization are demonstrated. Besides, we discuss some characteristics related to the poor prognosis caused by PGCCs in order to provide readers with a more comprehensive understanding of these huge cells.

## Introduction

Even though it is generally recognized that both prokaryotic and eukaryotic organisms are mostly diploid, organisms with more than two complete genomes have been widely observed. This kind of phenomenon, polyploid or whole genome duplication (WGD), is common for the ecosystem and is believed to have profound implications for evolution, especially in plant cells (1). In mammalian cells, blood megakaryocytes, hepatocytes, placental trophoblasts, and cardiomyocytes have all been proven to have polyploidization (2). As a classic mechanism throughout biological evolution, polyploidy plays an important role in normal development and differentiation, and in responding to adverse stimuli and injuries (2).

Not only in normal cells under physiological conditions, polyploid cells have also been found in tumor cells, which are considered to promote tumorigenesis and associated with adverse survival (3, 4). Many recent researches have indicated that stress, including X-ray or chemo-drugs, can induce polyploidization of malignant cells by causing mitotic cell cycle arrest and entering the endoreplication cycle, a more conducive process to adjust to the harsh living environment (5). These stresses can originate from the external environment, such as radiation, chemical drugs, and other treatments causing a fatal consequence of DNA double-strand break. It can also come from the terrible tumor microenvironment, such as inflammation, hypoxia, lack of nutrients, and overcrowding. When most of the stress-induced polyploid cells go to death, some of these cells have been observed to own ability of proliferation, accurately isolating the chromosomes required by diploid tumor cells from the chaotic genome and producing progeny cells through asymmetric cytokinesis, an unusual mechanism of depolyploidization (6). Since this kind of polyploid tumor cells were observed, they have been described by a variety of terms, with polyploid giant cancer cell (PGCC) being the most accepted (7). To summarize the opinions regarding to PGCCs in studies of recent years, what makes PGCCs differ from ordinary polyploid cells are stemness, dedifferentiation, the ability of repopulation, and particularly existing in posttreatment cancer. Meanwhile, other perspectives have suggested that the description of poly-aneuploid cancer cells is more accurate because chromosomes losing occurs frequently (8, 9).

In order to better present this emerging area of polyploid cancer research for readers, we summarized and discussed possible modes of tumor cell polyploidization under stress, different types of stress measures that lead to the polyploidization of tumor cells, and evidence of several different characteristics of PGCCs. Based on reviewing those knowledges, we hope to emphasis these special and small group of polyploid cells, and present a more comprehensive understanding of them.

## Several Mechanisms of Polyploidization

**Figure 1:**
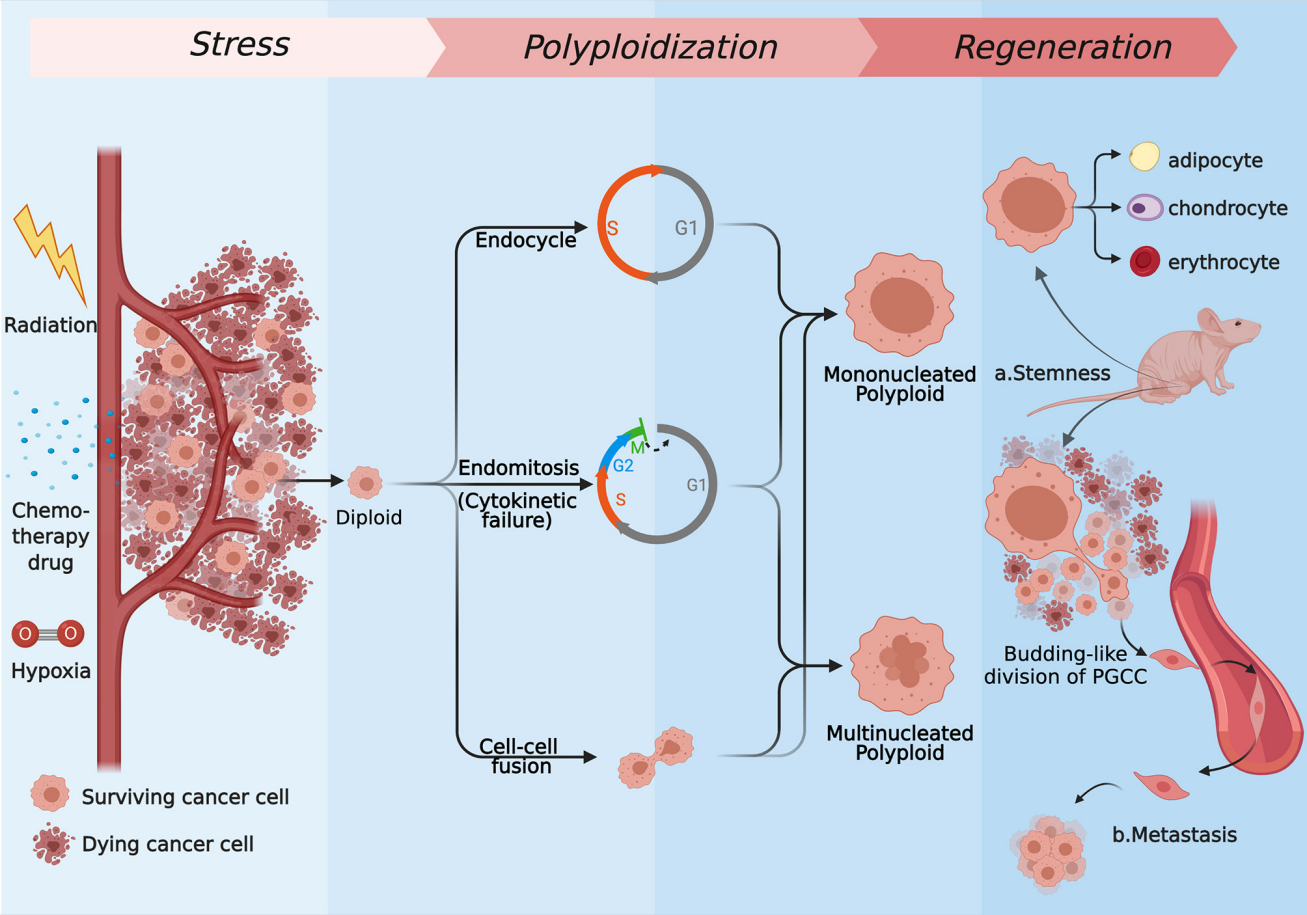
The several strategies of polyploidization under stress and the regeneration process of PGCCs. Most of the tumor cells are killed by radiation, chemotherapy drugs, hypoxia drugs, or other kinds of stress, while the rest of cells express resistance to those kinds of stress. Some of surviving cells form mononucleated or multinucleated cells through endoreplication (endocycle, endomitosis, or cytokinetic failure) or cell–cell fusion. Repopulating *via* neosis and differentiating to normal tissues like cells, PGCCs can show stemness *in vivo* **(A)**, and they are capable of generating distant metastases through the EMT **(B)**. (Created with BioRender.com).

In fact, as a means of response to stimuli, polyploidy, which has been noticed for a long time, presents wide applicability across species and cell types. Cells often need to differentiate to achieve permanent changes in their basic properties, satisfying a certain aspect of functional gain in this way (5, 10). Cells can defend lethal blows that can cause DNA double-strand break through increased cell volume and accumulated genetic material while enhance the transcription of metabolism and stress resistant genes (10, 11). This theory describes how polyploid cancer cells respond in cytotoxicity situations. For the sake of surviving in the rough-and-tumble context, the ways of tumor cells forming polyploid are diversified, and they occur to different degrees under the same background. The specific forms mainly include endoreplication (the first three modes) and cell–cell fusion (6, 12–15). First, forming polyploid cancer cells with a huge nucleus through endocycle is named mononucleated giant cells (MoNGC). Second, blocking another site of the mitotic cycle, the endoreplication mode to generate mononucleated giant cells is called endomitosis. Third, karyokinesis in mitotic cycle is accomplished normally when cytokinetic is blocked, which is described as acytokinetic mitosis or cytokinetic failure. These two kinds of situations are always regarded as the same, bringing about multinucleated giant cells (MuNGC). Fourth, fusion occurs between cells, which can be visualized as phagocytosis, entosis, or just evenly fusion. Those strategies are introduced in detail below (**Figure 1**).

### Endocycle: A Shortcut

The typical mitotic cycle consists of four consecutive periods, G1-S-G2-M. As a result, the chromatin in a diploid cell is replicated and averagely distributed, forming two diploid progeny cells after cytokinesis. Only when S phase-cyclin dependent kinase (S-CDK) and metaphase-cyclin-dependent kinase (M-CDK) are activated together, the total CDK formed by the two reaching a certain threshold, can the mitosis process be activated (16, 17). Especially in P53/Rb-deficient cells, by downregulating the level of M-CDK, which is responsible for driving G2/M phase process and the maintenance of S phase [composed of cyclin-dependent kinase-1 (CDK-1), cyclin A (cyc-A), and cyclin B (cyc-B)], the purpose of prolonging the G2 phase and preventing the cell cycle from entering the M phase can be achieved (15–18). At the same time, with the periodic inactivation of S-CDK (consist of CDK-2, Cyc-A, and Cyc-E), the G and S phases are driven alternately in pulses to allow tumor cells to enter endocycle, forming polyploid cells (15–18). That is to say, low level of the total CDK activation can maintain the endocycle (17). Thus, the endocycle can be understood as a breakaway from the ordinary cycle of mitosis, and a shortcut is established to form a small cycle inside the normal one. This small cycle connects the G2 phase with the G1 phase to form a simple cycle that only includes G and S phases and circulates back and forth between these two phases (5, 6, 16, 17). With neither dynamic changes of chromosomes nor division of nuclear membrane or cytoplasm, an enormous and polyploid nuclei containing large amounts of DNA finally comes into being (5, 6, 16, 17). In normal tissues, a classic case is the endocycle during wound healing in *Drosophila*. The injured *Drosophila* abdominal cells enter the S phase 24 h after injury, but there is no increase in the number of cells in the wound finally (19). Such mechanisms support organ growth and tissue homeostasis (15). As mentioned above, Tagal and colleagues believe that the possible mechanism for broad-spectrum or selective Aurora kinase inhibitors to promote the development of polyploid cancer cells in non-small cell lung cancer is endocycle. After withdrawal of AURKi, polyploid cancer cells have the ability to reverse to regular mitotic cycle (20).

### Endomitosis and Cytokinetic Failure: Depending on M Phase

Unlike endocycle, endomitosis has actually entered the mitotic stage and passed the G2/M checkpoint but has not completely finished the separation of sister chromatids and cytokinesis till the end (5). The final shape of the nuclei depends on the degree of completion of M phase, and we have known three situations. First, if the M phase of tumor cells is interrupted hurriedly before the anaphase stage and jump out of the mitotic cycle, a single giant nucleus cell can still be formed after multiple rounds of replication. Then, if the cell cycle passes the anaphase stage, without the complete nuclear division, the leafy nucleus cells can be made. Last, if mitosis is completed but cytokinesis is not performed, a polyploid containing multiple small nuclei is eventually caused, which is called acellular mitosis (or cytokinesis failure) (5, 14, 21, 22). In essence, the failure of cytokinesis can be regarded as a special kind of endomitosis, and they often discussed as the same phenomenon (21). Natural polyploid, megakaryocytes, in the blood system can provide an interesting idea. Its terminal maturation process involves multiple rounds of endomitosis and cytoplasmic restructuring to allow platelet formation (23). Continuous photographing of the fluorescence-labeled megakaryocytes under a microscope revealed that the megakaryocyte spindle successfully assembled and distributed the chromatid to the two daughter cells, which means that the dividing cells successfully passed the anaphase stage but reversed at the last moment of complete division (at this time, only some of the intercellular bridges formed by the spindles connecting to each other), and then, the two parts fused into polyploid cell, including the nucleus (24). Inhibition of cytokinesis activator, GTPase RhoA or its downstream pathway, RhoA/ROCK, activity can lead to decreasing aggregation of actin and myosin, resulting in the dysfunction of contractile loops required by mammalian cytokinesis and failure of cytokinesis (24–26). Meanwhile, Ganet et al. found that the active RhoA of tetraploid cells is lower than that of normal diploid cells (27). Apart from those, Aurora and polo-like kinases, whose dysregulation can lead to chromosomal mis-segregation and cytokinesis failure, are involved in the spindle assembly checkpoint (SAC) (21).

### Cell–Cell Fusion: More Direct Way

Aside from the abnormality of cell mitosis that brings about the generation of polyploid cancer cells, cell fusion between independent diploids is also a way to produce them, which has nothing to do with division. It is also the simplest and most direct idea to access to the way how polyploid cells are produced (28). In fact, cell fusion occurs in many normal tissues, such as *Drosophila* myoblasts (29), placental trophoblasts (30), hepatocytes (31), and fertilized eggs (32). The megakaryocytes described above also go through cell fusion after reversal mitosis. When different groups investigated the production mechanism of polyploid cancer cells, they all chose to label two kinds of tumor cells with different fluorescence and then cocultured both, observing mixed fluorescence to distinguish the fusion cells from the non-fusion cells (33, 34). For cells, the result of fusion is WGD and the accumulation of random mutations, which lay the foundation for the generation of aneuploid progeny in future multipolar divisions (28, 32, 34, 35). Aneuploid cells mean unequal distribution of genetic material, chromosomal instability (CIN), and more frequent DNA damage and mutation, undoubtedly aggravating the risk of malignant transformation, and spreading the abilities of drug resistance, metastasis, and stemness among malignant cells (34–36). It has also been recorded that merge cells produced by the fusion of tumor cells and normal cells [especially bone marrow-derived macrophages (37)] are more malignant because it is very possible for malignant cells to obtain transcription characteristics from normal cells, accompanied by chromosomal aberrations during fusion (such as translocations and inversions), so that any kinds of malignant polyploid cells created by this process that leads to tumor heterogeneity are not surprising (38, 39). An interesting clinical case can provide clues to the spontaneous cell fusion of tumor cells and normal cells in the body: a mother suffered from primary renal cell carcinoma 2 years after receiving bone marrow transplantation (BMT) from her son, and the Y chromosome was detected in kidney cancer cells, while her son remained healthy in the coming several decades (excluding donor source) (40). Although the author did not clarify vividly whether the cells produced by the fusion were more malignant, there are other studies to prove it. For example, the fusion cells of colorectal cancer cells with bone-marrow-derived blood macrophages can obtain extravasation, migration, immune evasion, and other capabilities, thereby enhancing the chance of metastasis (41). Melanoma cells can spontaneously fuse with macrophages or fibroblasts, making this process a rapid response mechanism to deal with environmental changes or even gain the ability of immune evasion (42). Breast cancer cells can activate epithelial–mesenchymal transformation (EMT) and Wnt/β-catenin signaling pathway by binding with macrophages to transform into invasive breast cancer cells (43). There are also studies about breast cancer that prove that the fusion of mesenchymal stem cells and breast cancer cells also enhance the migration ability of breast cancer cells (44).

Under severe stress, a large quantity of diploid tumor cells died, and the remaining cells formed polyploid cancer cells through the above-mentioned mechanisms. Studies and observations have shown that tetraploid cancer cells may produce aneuploidy through ploidy reduction and chromosomes loss, which reflect cytogenetically instability of cells with excessive chromosome numbers (45–48). The latest research has also shown that polyploid hepatocytes cause chromosomal instability during the process of ploidy reduction and also lead to an increase in the loss of tumor suppressor factors, thereby enhancing carcinogenicity (49). Tetraploid cancer cells in many researches are spontaneously produce in the process of tumor development, but we are more concerned about the polyploid cancer cells induced by treatment stress, whose number of genomes is far beyond the tetraploid. According to our experiences and a series of *in vitro* and *in vivo* observations, a small group of the stress-induced polyploid cells, which are so-called PGCCs, depolyploidize through a unique mechanism, neosis. Generally speaking, polyploid cancer cells can split into a limited number of progeny cells to reduce ploidy spontaneously, and gradually return to diploid, entering the mitotic cycle (4, 35, 45). Although appearing at the same time and same stress background as general polyploid cancer cells, PGCCs undergo karyokinesis *via* nuclear budding followed by asymmetrical division, directly producing many progeny cells only by PGCCs themselves in a short period (50). This powerful multiplication ability distinguishes PGCCs from normal polypoid cancer cells because one PGCC can quickly produce a large number of diploid or aneuploid progeny cells through this budding-like asymmetric cell division, causing tumor cell repopulation (50, 51). Thus, we would like to define PGCC by neosis, the special proliferation model.

PGCCs formed by endocycle and endomitosis have been observed in our *in vitro* system, while other researchers have observed that cell fusion may also play a part in the formation of these huge cells (33). Nevertheless, differences in formation mechanisms between PGCC and well-accepted polyploid cancer cell are largely unknown. As a new field of polyploid cancer research that is gradually gaining attention, different groups have tried to describe PGCCs from their own perspectives, which are associated with clinical practice. The stemness of PGCCs is often a classical direction, for this can be used to explain tumor recurrence in clinical patients (52–54). Some researchers focused on drug resistance of PGCCs (55), while other groups tried to find the molecular targets of PGCCs (56). These efforts can provide a research basis for the medication of tumor patients. Our group mainly paid attention to the tumor repopulation ability of PGCCs after stress (57). We believe that this model can simulate a pattern of tumor patients from first antitumor treatment to tumor recurrence.

## Stresses Leading to Polyploidization

The stress caused by treatment in clinical practice is the main source of external stimuli for tumor cells. Many *in vitro* evidence have shown that chemotherapy drugs, radiation, hypooxidative agents (CoCl_2_), mitotic inhibitors, and hyperthermia can all trigger endoreplication or cell fusion, leading to the production of polyploid tumor cells (**Table 1**). According to our experiences, slight stress, as small as ordinary cell passaging operation, could occasionally give rise to the formation of polyploid tumor cells. The hypoxia, nutritional deficiencies, temperature, and pH changes caused by these stresses continue to act on the proliferation and metabolism tumor cells, eventually leading to the initiation of this classic stress-responding mechanism.

**Table 1:** Several kinds of stress that can induce polyploidization of tumor cells.

Stimulus types	Cancer types	Cell types	Treatment	n	Evidence of polyploidy	Ref.
Chemotherapy	Colon cancer	HT29, HCT116, SW48, Caco-2	Trifluridine + Tipiracil	≥2n	DNA content analyze	(58)
		HCT116	Taxol, Vincristine	–	DNA content analyze	(59)
		LoVo, HCT116	Capecitabine, Oxaliplatin, Irinotecan	–	Morphological observation of cells, pathological sections of patients	(60)
		HCT116	Doxorubicin	≥4n	DNA content analyze, immunofluorescence, morphological observation of cells	(61)
		HCT116	Doxorubicin	4, 8, 16, 32	DNA content analyze, immunofluorescence, morphological observation of cell	(62)
		HCT116, SW480	Doxorubicin (DOXO), 5-fluorouracil (5-FU), Oxaliplatin (OXA), Irinotecan (IRINO)	–	Cell cycle analysis, immunofluorescence and morphological observation of cell	(63)
		HCT116	Cytochalasin D, Nocodazole, Docetaxel	4, 8, 16	DNA content analyze	(64)
	Ovarian cancer	Hey, SKOV3, OVCAR433	Paclitaxel	≥4n	DNA content analyze, immunofluorescence, morphological observation of cell	(12)
		Hey	Triptolide (TPL)	–	Morphological observation of cells	(65)
		Hey, SKOV3, MDA-HGSC-1	Paclitaxel	–	Immunofluorescence, morphological observation of cell	(66)
	Breast cancer	MDA-MB-436	Doxorubicin, Paclitaxel	–	Immunofluorescence, morphological observation of cell	(67)
		BT-549	Triptolide (TPL)	–	Morphological observation of cells	(65)
		MDA-MB-231	Nocodazole	4, 8	DNA content analyze, morphological observation of cells	(68)
		MDA-MB-231	Doxorubicin	4, 8	Immunofluorescence, morphological observation of cell	(69)
		MDA-MB-231, MCF-7	Doxorubicin	–	Immunofluorescence, morphological observation of cell	(70)
		MDA-MB-231	Doxorubicin	4–128	DNA content analyze, immunofluorescence, morphological observation of cell	(71)
	Prostate cancer	PC-3	Docetaxel	≥4n	DNA content analyze, immunofluorescence, morphological observation of cell	(55)
		PC-3	Docetaxel	–	Morphological observation of cells	(72)
		PPC1	Docetaxel	–	Morphological observation of cells, flow cytometry	(56)
	Prostate cancer	PC-3	Docetaxel	–	Immunofluorescence, morphological observation of cell	(73)
		Du-145	Docetaxel		Immunofluorescence, morphological observation of cell, flow cytometry	(74)
	Lung cancer	H1299	Chemptothecin, Doxorubicin, Cisplatin	4, 8, 16	DNA content analyze	(75)
		A549	Cisplatin	–	Morphological observation of cells, flow cytometry	(56)
	Glioblastoma	LN-18	Doxorubicin	4, 8, 16, 32	DNA content analyze, immunofluorescence, morphological observation of cell	(62)
		U87, A172, U251, U138	Temozolomide (TMZ) Vinblastine (VBL), Mebendazole (MBZ)	–	Cell cycle analysis	(76)
	Lymphoblastoma	WIL2-NS	Paclitaxel (PTX)	4, 8	Immunofluorescence, morphological observation of cell	(69)
	Liver cancer	HGC-27	Cisplatin + Paclitaxel (C+P), Cisplatin+ Paclitaxel+ Docetaxel (C + P + D)	≥4n	DNA content analyze, immunofluorescence	(77)
		Hep-G2	Cytochalasin D, Nocodazole, Docetaxel	4, 8, 16	DNA content analyze	(64)
	Chronic myeloid leukemia	K-562	Cytochalasin D, Nocodazole, Docetaxel	4, 8, 16	DNA content analyze	(64)
Radiotherapy	Cervical cancer	Hela	10 Gy	4–64	Immunofluorescence, morphological observation of cell	(69, 78)
	Colon cancer	LoVo, HCT116	9 Gy	–	Morphological observation of cells, pathological sections of patients	(60)
	Prostate cancer	PPC1	8 Gy	–	Morphological observation of cells, flow cytometry	(56, 79)
	Melanoma	MEL624-28	8 Gy	–	Morphological observation of cells, cell cycle analysis	(79)
	Glioblastoma	U118	12 Gy	–	Morphological observation of cells, cell cycle analysis	(79)
	Breast cancer	MDA-MB-231	6, 10, 14 Gy	–	Immunofluorescence, morphological observation of cell, cell cycle analysis	(57)
	Lung cancer	A549	8, 10 Gy	–	Morphological observation of cells, flow cytometry	(56)
	Lymphoblastoma	WIL2-NS	10 Gy	4, 8	Immunofluorescence, morphological observation of cell	(69)
Aurora kinases inhibitors	Colon cancer	HCT-116	VX-680, AZD1152-HQPA, MLN8237	4, 8, 16	DNA content analyze, immunofluorescence, morphological observation of cell	(80)
	Acute myeloid leukemia	HEL, GDM-1, MOLM-13	AMG-900	4, 8, 16	DNA content analyze, immunofluorescence, morphological observation of cell, cell cycle analysis	(81)
	Liposarcoma	SW-872, 93T449	AMG 900, AZD1152-HQPA, MK-5108	4, 8, 16	DNA content analyze, immunofluorescence, morphological observation of cell	(82)
	Oral squamous cell carcinoma	CAL27	Tan IIA	–	Immunofluorescence, morphological observation of cell, cell cycle analysis	(83)
	Lung cancer	NCI-H1693	MLN8237, VX-689, AZD1152, GSK1070916	–	Immunofluorescence, morphological observation of cell	(20)
Hypoxia	Breast cancer	MCF-7, MDA-MB-231	CoCl_2_	–	Morphological observation of cells, pathological sections of patients, cell cycle analysis	(84)
		MDA-MB-231			Immunofluorescence, morphological observation of cell, cell cycle analysis	(33)
	Ovarian cancer	Hey			Morphological observation of cells, pathological sections of patients	(85)
		Hey, SKOV3			Immunofluorescence, morphological observation of cell, cell cycle analysis	(33)
	Colon cancer	HCT-116, Caco-2			Immunofluorescence, morphological observation of cell, cell cycle analysis	(86)
Hyperthermia	Colon cancer	HCT-116	42°C	4, 8	Immunofluorescence, morphological observation of cell, cell cycle analysis	(87)

In our previous studies, it was proved that the irradiation dose of 10 Gy could induce polyploidy in MDA-MB-231 and HeLa cells. As the dose increased from 6 to 14 Gy, the proportion of polyploid cells rose from 6.09% to 34.79%, while some polyploid cells participated in the regeneration of diploid cells (57). This proportional change indicated that as the stimulation level continued to intensify, the number of polyploid cells in the population increased. In other words, it is the intensity of stimulation that directly determine the population size of these giant nucleus cell. At the same time, the polyploidization of tumor cells induced by radiation is not unique to any specific cell line. In addition to cervical cancer and breast cancer cells, many others, such as lung cancer, colon cancer, melanoma, prostate cancer, glioblastoma, and lymphoblastoma, have been demonstrated *in vitro* to produce polyploid cells after irradiation, revealing the universality of this mechanism under the effect of X-rays. In the follow-up studies, we focused on those polyploid cells induced by irradiation and found that a fraction of these cells, which were usually considered to be senescence or mitotic catastrophe, did not simply die. Instead, they expressed extraordinary proliferation ability through an unclear, non-meiosis, or mitotic method to de-polyploid (57). This process was first given by Rajaraman and his colleagues a professional term—neosis (50). We believe that it is neosis that represents the most important feature among many characters of PGCCs, for it mediates the repopulation of minimal residual disease after antitumor therapy. In fact, as early as 1956, Puck has discovered that a single Hela cell can be induced to polyploidization after radiation treatment and form new clones by producing progeny cells that are morphologically indistinguishable from untreated cells (88). Many subsequent studies following Puck, in which a part of radiation-induced polyploid cells undergo genetic disorder and depolyploidization to produce diploid or aneuploid cells, have been repeatedly verified (78). White-Gilbertson and his colleagues found that the expression of acid ceramidase (ASAH1) is elevated in PGCC induced by radiation from both prostate cancer and lung cancer (56). In their other research, although ASAH1 inhibitor tamoxifen could not block the formation of PGCC under the action of therapeutic stress, it prevented the process of neosis (79).

Using radiation to treat tumor cells *in vitro* to induce the formation of polyploid cells has been one of the mature modes that are often utilized to simulate the response of postradiotherapy tumor patients in experimental research. Furthermore, treating tumor cells with chemotherapeutic drugs simulates another irreplaceable method of clinical tumor therapy. Compared with the working principle of radiation, the pharmacological effects of different chemotherapeutics are quite disparate, so that they are more favored by researchers, including Gilbertson, who also used docetaxel as another operation to induce polyploid cells in parallel with the radiation in his research (56). Among all kinds of cancers in human being, colon cancer, breast cancer, prostate cancer, and ovarian cancer are the most attractive ones in the studies of response to chemotherapy drugs. Niu and other researchers applied gradient concentrations of paclitaxel to treat different types of ovarian cancer cells and found that within the range of 0–500 nM, as the number of dead cells soared, PGCCs increased the most at the top concentration (few cells survived in higher concentration) (12). Lin et al. employed docetaxel to induce polyploid cells in their research, which further clarified the relationship between stress intensity and the production tendency of polyploid cells (73). In the concentration range below 2 nM, few PGCCs were induced, while the yield of PGCCs in the range of 2–4 nM showed a rapid increase trend, and PGCC was even the only survival cell type in the concentration range above 4 nM (73). This phenomenon indicates that within a certain range, as the stimulation of chemotherapeutic drug continues to rise, the number of PGCCs grows in proportion to the stimulation strength. Meanwhile, Niu and other researchers observed multiple progeny cells being separated from PGCCs through asymmetric division by live-cell fluorescence time-lapse recording technology (12). Moreover, it has been documented that Docetaxel induces prostate cancer cells to produce polyploid cells, and DNA can be passed into progeny cells through the small branches of multinucleated polyploid (55). By verifying the discrepant expression level of cleaved caspase-3 after drug treatment between multinucleated polyploid cells with their progeny and control PC-3 cells, researchers proved that the former two have less apoptotic ratio and better drug resistance (55).

Several recent studies have focused on Aurora kinases (AURK). A variety of broad-spectrum or specific Aurora kinases inhibitors (AURKi), including inhibitors targeting on three highly conserved serine/threonine kinases, Aurora A–C, respectively, have been confirmed to induce polyploid cells by regulating mitosis or cell division arrest (80–83). Since the expression of Aurora kinases, especially Aurora A and B, on centrosomes and chromosomal centromeres is essential for mitosis (89, 90), it has received widespread attention that AURK inhibitors have the potential to become a therapeutic target to bring about tumor cell senescence and death. Despite this, a fresh experimentation illustrated that there are always drug-resistant polyploid cells surviving, whether using wide-spectrum inhibitors or applying selective AURKi together or apart, which has a stronger surviving property in the combat with antimitosis drug, like paclitaxel and docetaxel (20). The tough living situation forces tumor cells to achieve the cycle of parental diploid–polyploid–diplontic progeny through depolyploidization. Interestingly, the offspring of diploids acquired a new phenotype of drug resistance while maintaining the same proliferation ability (raised expression of antiapoptotic protein BCL-2 and survivin (55). The heritability of the new phenotype has not been continuously verified. If it can be stably inherited to later generation through mitosis, the cell line has evolved under the push of antimitosis operations.

Apart from the attack measures that directly generate cytotoxicity, the stress response induced by changes in the tumor microenvironment that leads to the emerging of PGCCs has also been affirmed. A widely accepted method is proposed by Liu, Zhang, and their colleagues, who added CoCl_2_ to the culture medium to simulate the hypoxic environment, thereby activating the intracellular hypoxic pathway. The feasibility of this approach has been tested in many cell lines of ovarian cancer (33, 85), breast cancer (33, 84), and colon cancer (86). There is also laboratory evidence that intermittent blow at 42°C can cause centrosomes and spindles dysfunction in colon cancer cells HCT116 with stable karyotype (87). Besides, real physical strain is also a bad stimulus for cells. The established microgravity device has simulated the strong stress of the cells under the tissue fluid inside the tumor and proved the production of PGCCs induced by physical pressure (91).

## Certain Characteristics of Giant Cells

### PGCCs Exhibit CSC-Like Feature

PGCC has the ability to recover from a single polyploid cell to a colony, while cancer stem cells (CSCs) are considered to be the culprit that mediate tumor repopulation after treatment and lead to disease relapse. Those two are quite similar, so whether PGCCs expressing stem cell-like properties has also attracted the attention of many researchers. In “the dualistic origin” theory he created, Liu has ingeniously compared and unified the PGCC proliferation cycle (giant cell cycle) with the embryonic development cycle (6, 92). This theory not only provides a physiological basis for the development of PGCC but also reveals how mature somatic cells differentiate and re-express stemness under the action of stress (6, 92). The current research evidence of PGCC with stem cell characteristics can be roughly divided into three aspects. First, it has surprisingly strong proliferation ability. Second, the big cells can be induced to differentiate into some normal tissues. Finally, PGCCs have been shown to express stem cell markers. By sorting single multinucleated tumor cells and injecting them under the skin of nude mice, Weihua and colleagues successfully constructed a single PGCC-level *in vivo* tumor model (93). This working method makes their study a classic evidence of PGCC’s powerful tumorigenesis ability. Apart from this, PGCC also has the potential for multidirectional differentiation and plasticity in differentiation types. In Zhang’s laboratory, they repeated the precise experiment to verify the high tumorigenicity of PGCCs, and another important achievement of theirs also showed that the use of special medium can induce PGCC to differentiate to several types of mesenchymal tissues, such as adipose, cartilage, and bone (33). This finding was also confirmed by *in vivo* modes. His two other studies have displayed that PGCCs also worked out erythrocyte-like cells and embryonic hemoglobin with strong oxygen binding ability (94, 95). This makes a positive impact on improving the hypoxic environment inside the tumor tissue. A series of stem-cell-specific markers are also ideal indicators for detecting the stemness of PGCCs. Recognized as CSC markers, CD44 and CD133 have been listed by many laboratories in exploring PGCC’s stem-cell-like properties (33, 54). The high expression of OCT4, SOX2, and Nanog in polyploid cells also reveal their multidifferentiation potential and self-renewal ability similar to embryonic stem cells, and this expression pattern can be passed on to the diploid progeny cells they produce (96, 97). In later studies, Niu et al. proved that the expression of these three markers in PGCCs is time and space dependent (66). An investigation carried out by David et al. focused on pregnant cells, which were very similar to the PGCC discussed here. It is interesting that although these cells expressed stem-like phenotype physiologically, they seldom expressed CSC-related antigen markers (98). Meanwhile, in their progeny cells, especially the first generation of progeny cells, high levels of CSC markers, such as CD44, CD133, OCT4, and Nanog, were detected (98). Based on the above research clues, whether the proliferation mode of neosis presented by PGCC is the same as that of CSC still remains to be studied. There is no doubt, however, that the progeny diploid cells produced by PGCCs, which are very likely to participate in tumor recurrence after treatment, express many stem markers and are extremely malignant.

### Progeny Cells Dominate Tumor Metastasis

In the experiment of Weihua with a single PGCC to establish an *in vivo* subcutaneous tumor model, except tumor formation *in situ* inoculation, they observed the formation of metastases in the lungs of some nude mice, too (93). This phenomenon released a clear signal about the metastatic ability of PGCCs with their progeny cells. Zhang’s team has done a set of studies on the metastatic ability of this cluster. Comparing the invasion and migration ability of PGCCs with daughter cells in breast cancer cell and the control one, it was found that the invasion and migration ability of the formal group was much more powerful, accompanied by metastasis-related markers, N-cadherin and vimentin, significantly going up (84). By inhibiting the expression of S100A4 in another one, the downstream cathepsin and cytoskeletal protein functions were disturbed, thereby weakening the invasion and metastasis ability of PGCCs and its progeny cells (99). However, whether PGCCs themselves having metastasized or the progeny cells produced by neosis being more aggressive have not yet been carefully discussed. We believe that the accumulation of DNA in stress-induced polyploid cells is a response mechanism, and its progeny cells are something like rescue capsules of PGCCs after chromosomal disorder, which may lead to a more aggressive and stress-adaptable phenotype. In a study after multiple subcultures of PGCC, it was shown that after passaging to the 10th generation (P10), the invasion and metastasis ability was obviously stronger than that of the primary generation and even stronger than that of untreated control cells (65). Although this experiment did not directly isolate the offspring, the proportion of progeny cells gradually grown up as the number of passages climbed, which can also indirectly illustrate the important role of progeny cells in metastasis. Another phenotype closely related to metastasis involved in the above studies is EMT. Multiple studies have involved evidence that PGCCs and progeny cells upregulate the expression of some EMT-related markers, including the raising of ZEB1 in prostate cancer PGCCs (73); the expression of Twist, Slug, and Snail building up in colon cancer PGCCs (95); and the enhancement of N-cadherin, vimentin, and cathepsin in PGCCs of a few cancer cell lines (84, 95, 99). Listing evidence of PGCCs’ stemness, we mentioned that they could be induced to differentiate into a variety of mesenchymal tissues (33). Apart from participating in embryo differentiation and wound healing as an indispensable physiological process, EMT can also make epithelial cancer cells lose their polarity, confer them mesenchymal properties, and allow them to transform or stay between two extreme states arbitrarily (100, 101). Hence, EMT and MET are two dynamic mutual changes rather than two states of fragmentation (102). The EMT process can cause cancer cells to metastasize to a distant place and then restore the epithelial state through the MET process to form tumor (103). In short, the EMT mechanism cannot only make PGCCs show the relevant characteristics of tumor stem cells but also play a role in the metastasis of tumor cells, thereby linking stemness and metastasis in PGCC.

### PGCCs Behave Numerous Senescent Phenotypes

Previously, it was believed that polyploid cells were the result of mitotic catastrophe and lost the ability of cell proliferation. As a matter of fact, the two key characteristics of senescent cells are cell cycle arrest and degradation of proliferation ability (104). This makes the discussion of PGCC always be accompanied by its relationship with the senescence phenotype, just as a couple of recent reviews systematically introduced the complex connections and differences between polyploidy and senescence (105–107). A handful of common features between polyploidy and senescence were summarized, including induction of DNA damage, activation of P53/Rb, escaping from cell death, growing incidence of autophagy, common associated genes, and recovery of stemness gene (107). Senescence was generally regarded as a powerful antitumor mechanism in the past (108, 109). If the two do have a lot in common, this is confusing when many views hold the point that polyploid cells are involved in tumor recurrence (9, 13). Sikora and colleagues discussed therapy-induced senescence (TIS) and therapy-induced polyploidy (TIP), unifying their roles in tumor DNA damage response mechanisms and clarifying that senescence and polyploidy are two necessary conditions for neosis to play a role in the repopulation of PGCCs, especially the later one (105). In other words, senescent cells that have failed to undergo polyploidization may not be able to escape the fate of senescence and death in the end, neither returning to the mitotic cycle (105). This view can explain the recent research on the restoration of proliferative activity of senescent cells induced by antitumor stress (110). The cells that escape the fate become more adaptable to stress, more heterogeneous, and more aggressive (111).

### Several Meiotic Genes Are Activated

As we all know, there are two processes of reduction in ploidy in the normal division of mammals: the separation of sister chromatids into two daughter cells in the late mitosis period, resulting in the transformation from tetraploid to diploid, and two consecutive ploidy reductions in the process of meiosis, from tetraploid to haploid. It is documented that structures similar to meiotic synaptonemal complexes were observed in polyploid cells 7–9 days after treating Hela cells with 10 Gy rays (112). It was also detected that the expression of meiosis-specific genes SYCP, REC8, and DMC1 went up in cervical, breast, and colon cancer cells after irradiation (112). Another study on meiotic adhesive REC8 showed that REC8 changed its location from the centromere to the centrosome and astral fiber after irradiation, and colocalized with the microtubule-associated protein to induce chromosomes segregation and genome reduction in polyploid cells (113). A recent research holds the view that under the action of meiotic nuclease SPO11 and its downstream Mos-kinase, meiotic and telomere-related mechanisms may play a role in the neosis of PGCCs (71). Interestingly, some of the aforementioned meiosis-specific genes in radiation-induced polyploid cells were first time to be found in malignant tumor cells (114). In the proliferation mode of neosis, there is a process by which polyploid actively reduces and eliminates excess chromatin. It is not surprising that this process involves the initiation of meiosis-specific genes. More importantly, whether these genes are likely to become therapeutic targets and how PGCCs can accurately screen and repair the specific number of genes required for offspring diploids from a group of chaos are the focus of attention in the future.

## Conclusion

**Figure 2:**
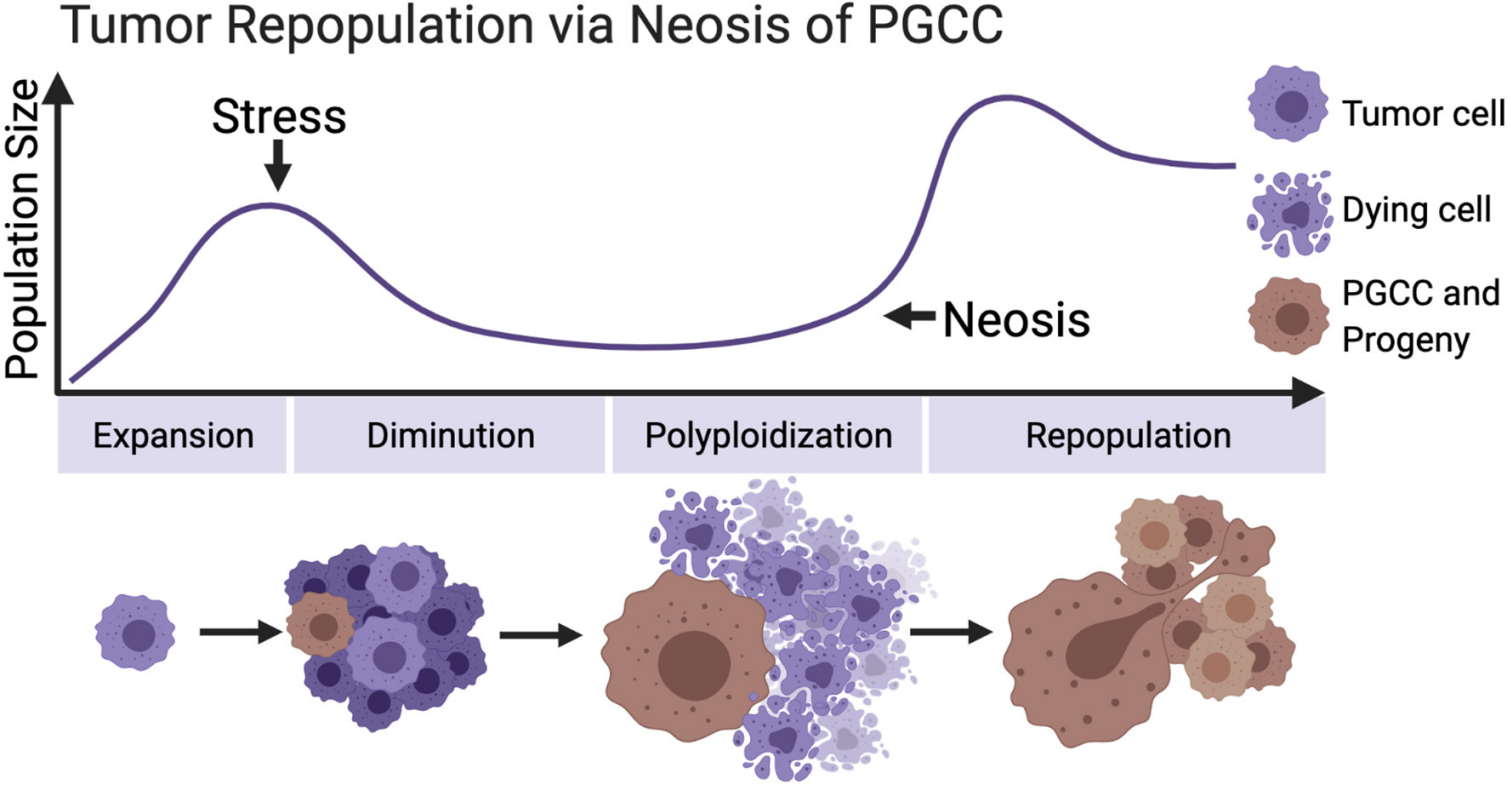
Primitive model of tumor repopulation *via* neosis of PGCCs. When tumor cells proliferate to a certain scale for the first time, they encounter treatment stress or other kinds of survival pressure. Most of the tumor cells are killed, while a small part of them survive and form PGCCs, containing the extensive potential to re-enter the proliferation cycle through neosis. (Created with BioRender.com).

The stresses that can induce tumor cells to form polyploid are diverse and cover the current radiotherapy and chemotherapy commonly utilized in clinical tumor treatment. These stimuli cause the death of a large quantity of tumor cells and promote the formation of PGCCs in the meantime. These polyploid cells neither lose the ability to proliferate nor wait for death. The unique asymmetric division mode—neosis—mediates the process of tumor repopulation (**Figure 2**). Moreover, the strong adaptability, stem cell characteristics, metastasis, and senescence escaping expressed by PGCCs all reveal that they are closely related to posttreatment relapse. These malignant cells must be included in the scope of the key target in future tumor therapy plan.

As a newly emerging field, there are still many questions that need to be resolved. Although many morphological and pathological descriptions of PGCCs have been carried out, and some theories related to the life cycle of these cells have been proposed, there are still many pivotal problems that need to be followed and solved. First of all, are there any differences in the mechanisms of polyploidization between PGCCs and well-accepted polyploid cancer cells? If it does, then what drives the two express different phenotypes under the same stress. If not, researches should look for a new angle to establish the fate spectrum of polyploid cells, such as molecular markers. Second, as described in the first question, there is still a lack of mature molecular markers as screening and therapeutic targets. Furthermore, most of the work on PGCCs was done on immortal cell lines *in vitro*. Although there are some pathological evidence about PGCCs *in vivo*, it is still necessary to show production and proliferation of PGCCs *in vivo* more intuitively. Finally, we believe that the specific ploidy-reducing mechanism of PGCCs is of vital importance, for its role in the repopulation of minimal residual disease of tumor should not be overlooked. At present, scientists should continue to focus on exploring the special proliferation mode of PGCCs and the molecular marks related to it, so as to pick out molecular targets that can be transformed into clinical application.

## Author Contributions

YS, YZ, ZD, and QH contributed to the conception of the study. YS, YZ, ZD, and RZ performed the document retrieval. YS wrote the manuscript. All authors contributed to the article and approved the submitted version.

## Funding

This study was supported by a funding from National Natural Science Foundation of China (No. 81972843 to QH).

## Conflict of Interest

The authors declare that the research was conducted in the absence of any commercial or financial relationships that could be construed as a potential conflict of interest.

## Publisher’s Note

All claims expressed in this article are solely those of the authors and do not necessarily represent those of their affiliated organizations, or those of the publisher, the editors and the reviewers. Any product that may be evaluated in this article, or claim that may be made by its manufacturer, is not guaranteed or endorsed by the publisher.
